# Xeno-Free Materials for Stabilizing Basic Fibroblast Growth Factor and Enhancing Cell Proliferation in Human Pluripotent Stem Cell Cultures

**DOI:** 10.3390/ma15103687

**Published:** 2022-05-20

**Authors:** Yoko Masuzawa, Manabu Kitazawa

**Affiliations:** Institute for Innovation, Ajinomoto Co., Inc., 1-1 Suzuki-cho, Kawasaki-ku, Kawasaki City 210-8681, Japan; m-kita2012@cg.em-net.ne.jp

**Keywords:** basic fibroblast growth factor, induced pluripotent stem cells, sulfated materials, heparin, polysaccharides, synthetic polymers

## Abstract

Induced pluripotent stem cells (iPSCs) are widely considered important for developing novel regenerative therapies. A major challenge to the growth and proliferation of iPSCs is the maintenance of their undifferentiated status in xeno- and feeder-free conditions. Basic fibroblast growth factor (bFGF) is known to contribute to the expansion of stem cells; however, bFGF is notoriously heat-labile and easily denatured. Here, we investigate the effects of a series of synthetic sulfated/sulfonated polymers and saccharides on the growth of iPSCs. We observed that these materials effectively prevented the reduction of bFGF levels in iPSC culture media during storage at 37 °C. Some of the tested materials also suppressed heat-induced decline in medium performance and maintained cell proliferation. Our results suggest that these sulfated materials can be used to improve the expansion culture of undifferentiated iPSCs and show the potential of cost effective, chemically defined materials for improvement of medium performance while culturing iPSCs.

## 1. Introduction

Induced pluripotent stem cells (iPSCs), first reported in 2006 for mice [1] and in 2007 for humans [2,3], are expected to play an important role in new regenerative medicine therapies [1,2,3]. Regenerative medicine using iPSCs requires efficient methods for establishing and expanding cell cultures [4,5,6], while maintaining their undifferentiated status. Considerable research is therefore being conducted [7,8]. It has been reported that iPSCs can be stably established and expanded in feeder- and xeno-free conditions [9]; however, the further development of economic and efficient culture methods is still warranted, as culture media are among the key components.

Basic fibroblast growth factor (bFGF) has been shown to be effective in improving cell proliferation while maintaining undifferentiated cell status in the expansion cultures of embryonic stem cells (ESCs) and iPSCs through activating signaling pathways, such as mitogen-activated protein kinase (MAPK), phosphatidylinositol-4,5-bisphosphate 3-kinase (PI3K)/AKT, phospholipase C gamma (PLCγ) and signal transducers and activators of transcription (STAT) [10,11,12,13]. As a multifunctional protein with mitogenic activity, bFGF also has regulatory, developmental, and endocrine effects [14,15]. However, bFGF is known to be heat-labile and prone to denaturation [16,17]. Culture media containing bFGF are therefore recommended to be stored at low temperatures and used within 1 week. The stabilization of bFGF has been extensively studied for pharmaceutical applications [18,19,20]. In a previous study, we investigated the effects of sulfated materials for the stabilization of bFGF in human mesenchymal stem cell (hMSC) culture media; some of the effective compounds for bFGF stabilization also promoted hMSC proliferation [21].

Although many approaches to the stabilization of bFGF in iPSC culture have been tested, their suitability for practical application remains to be assessed [22,23]. Various iPSC culture methods, including good manufacturing practice (GMP)-compliant automated production techniques, have also been studied for practical use [24,25,26,27,28]. The media often deteriorate, owing to the culture process itself or frequent temperature fluctuations caused by culturing and refrigeration. As this is a common issue for culture media, it is recommended that media be used within 1 week. The economic efficiency of media can be improved if media performance can be preserved for longer periods. Recently, novel media, such as mTeSR-Plus and StemFlex, which contain bFGF-stabilizing materials, have become commercially available, and such media may enable stable cultures and easy operations without any weekend feedings. The bFGF-stabilizing materials in these media have not been disclosed [29]. FGF signaling involves its binding to receptors along with proteoglycans, such as heparin, which is known to stabilize bFGF. The addition of heparin has been reported to promote the growth of ESCs [30]. However, the heparin available on the market is of animal origin and is not chemically defined.

We investigated simple and chemically defined supplements to prevent bFGF deactivation and promote cell proliferation. Adding simple, chemically defined supplements bearing sulfate/sulfonate groups has been shown to protect bFGF from heat-induced deactivation in hMSC media, resulting in enhanced cellular growth [21]. In this study, we investigated the efficacy of such materials in human iPSC culture. We used enzyme-linked immunosorbent assays (ELISAs) to evaluate the protective effects of various compounds against the decrease of bFGF when added to a commercial iPSC culture medium and stored for 1 week at 37 °C. We also investigated the effects of these materials on iPSC proliferation. The addition of the test materials effectively suppressed the deterioration in medium performance caused by the loss of bFGF. Our results revealed the possibility of improving the procedure for the expansion culture of iPSCs by exclusively using xeno-free materials.

## 2. Materials and Methods

### 2.1. Reagents and Chemicals

iPS Academia Japan, Inc. (Kyoto, Japan) provided the hiPSC line 201B7. Nippi (Tokyo, Japan) provided Laminin511-E8 (iMatrix-511); Corning (New York, NY, USA) provided Matrigel^®^; and Ajinomoto Co., Inc. (Tokyo, Japan) provided StemFit^®^AK02N medium. Thermo Fisher Scientific (Waltham, MA, USA) provided Essential 8™ (E8) medium, Dulbecco’s modified Eagle’s medium (DMEM), ethylenediaminetetraacetic acid (EDTA) solution, and TrypLE™ Select CTS™. Nacalai Tesque, Inc. (Kyoto, Japan) provided Y-27632 (catalog#:08945-84) and Hoechst staining solution (catalog #:33258) as well as heparin sodium and polyallylamine. Sigma-Aldrich (St. Louis, MO, USA) provided penicillin-streptomycin (100 U/mL, 100 μg/mL), albumin (from human serum, A1887), leukocyte alkaline phosphatase kit (catalog #:86R), polyvinyl sulfonate, and sulfur trioxide trimethylamine complex. Fujifilm Wako Chemicals (Tokyo, Japan) provided dextran sulfate sodium (MW 5000 Da, sulfur content 15–20%) and maltoheptaose, while Funakoshi (Tokyo, Japan) provided fucoidan. Tokyo Chemical Industry (Tokyo, Japan) supplied alginate, xanthan gum, pectin, and maltotriose; Merck (Darmstadt, Germany) supplied cellulose; Polyscience (Warrington, PA, USA) supplied polyvinylamine (MW 25,000 Da); Carbosynth (Compton, UK) supplied sucrose octasulfate potassium; Acros Organics (Belgium) supplied polyvinylalcohol (PVA) (MW 16,000 Da, 98% hydrolyzed); and Kanto Chemical (Tokyo, Japan) supplied dimethylformamide.

### 2.2. Synthesis of Sulfated Materials

PVA sulfate was prepared using a method described previously [21]. Briefly, PVA (200 mg; MW 16,000 Da, 98% hydrolyzed) was dissolved in 600 mg sulfur trioxide trimethylamine complex in dehydrated N,N-dimethylformamide (6 mL), and the mixture was incubated overnight at 70 °C with stirring. The supernatant was removed through decanting, and acetone (20 mL) was added to the precipitate. After stirring, the precipitate was collected by filtration, and the resulting solid was dissolved in distilled water (2 mL). Subsequently, 1.5 mL of 30% aqueous sodium acetate was added, and the mixture was stirred for 2 h at 25 °C. The precipitate was collected by filtration and again dissolved in distilled water (3 mL) after adding 12 mL of ethanol to this mixture. Dialysis tubing (Spectra/Por MWCO 6000–8000; Spectrum Laboratories, Bruker Ultrashield, CA, USA) was used to dialyze the solution overnight against distilled water, and the dialysate was lyophilized to obtain 61 mg of a white solid. Using ^1^H-nuclear magnetic resonance (NMR) spectroscopy at 400 MHz, the chemical composition and structure of the obtained compound were confirmed (Bruker Ultrashield, Bruker, Billerica, MA, USA). Inductively coupled plasma emission spectroscopy was used to determine the sulfur content of the molecule (ICPS-8100, Shimadzu, Kyoto, Japan).

The other sulfated/sulfonated compounds were synthesized in the same way, with minor differences. For 200 mg of saccharides, 1 g of sulfating reagents and 10 mL of solvent were used to synthesize sulfated polysaccharides (xanthan gum sulfate, alginate sulfate, pectin sulfate, and cellulose sulfate) and oligosaccharides (maltoheptaose sulfate and maltotriose sulfate). The amounts of 2.5 g of sulfating reagents and 25 mL of solvents were used for 200 mg of synthetic polymers (polyvinylamine sulfonate and polyallylamine sulfonate). Dialysis tubing (MWCO 1000) was employed to purify the oligosaccharides. NMR and ICPS were used to confirm the chemical compositions and structures of all the compounds.

### 2.3. Measurement of bFGF Concentration in Culture Media Using ELISA

The concentrations of bFGF in the E8 media were determined according to the manufacturer’s instructions using a human bFGF ELISA kit (RayBiotech, Peachtree Corners, GA, USA). Using a microplate reader, the absorbance of the ELISA samples was measured at 450 nm (Corona Electric, Ibaragi, Japan). The smallest level of human bFGF that could be detected was 50 pg/mL. All samples were prepared in triplicate and then analyzed.

### 2.4. Evaluation of Test Compounds Using ELISA

Phosphate-buffered saline (PBS) was used to dissolve the test substances (heparin, dextran sulfate, fucoidan, alginate sulfate, cellulose sulfate, xanthan gum sulfate, maltoheptaose sulfate, maltotriose sulfate, sucrose octasulfate, PVA sulfate, polyvinyl sulfonate, polyvinylamine sulfonate, and polyallylamine sulfonate) at a concentration of 25 mg/mL. They were then diluted to a concentration of 2.5 mg/mL in the culture medium. The required final concentrations (250 pg/mL–2.5 μg/mL) were then achieved by ten-fold serial dilutions with the medium. In sealed Falcon tubes, test samples were incubated at 37 °C for 7 days before being stored at 4 °C overnight. The concentrations of bFGF were then determined using an ELISA kit. On the day of preparation, osmotic pressure and pH were recorded and pH was measured again on the 7th day to ensure that no substantial changes had occurred [21]. All samples were prepared in triplicate and analyzed.

### 2.5. Precoating of Culture Substrates

Precoating and cell culture conditions followed a previous report [31]. All extracellular matrix (ECM) coating solutions were prepared according to the manufacturer’s instructions. Prior to cell seeding, wells were coated with iMatrix-511 or Matrigel at concentrations of 0.25–1 μg/mL and stored overnight at 4 °C. The matrix solution was diluted by 1:100 in DMEM to make Matrigel. The ECM solution was coated with concentrations of 8.3–12.5 μg/cm^2^ of Matrigel or 0.5 μg/cm^2^ of iMatrix-511 and stored overnight at 4 °C prior to cell seeding. The coating solution volume was adjusted based on the growth area of the culture vessels employed.

### 2.6. Cells and Culture Conditions

We followed the Center for iPS Cell Research and Application’s standard instructions for transferring hiPSCs from a feeder to a feeder-free culture [9]. The hiPSCs were employed at 3–10 passages after being cultivated and 36–45 passages after being established. In the Stem Fit AK02N medium, the hiPSCs were kept in feeder-free conditions on ECM protein-coated surfaces. The cells were treated with a detachment solution, which contained 0.5× TrypLE™ Select CTS™ containing 0.25 mM EDTA solution, for 8 min at 37 °C for single cell passaging. The vessel was filled with culture medium containing 10 mM Y-27632, and single-cell suspensions were generated by pipetting numerous times before cell counting. The trypan blue exclusion test was used to determine the number of viable cells in the culture vessels, which was evaluated using the automated counting of detached cells from 50 representative images (Vi-CELL™ XR, Beckman Coulter, Brea, CA, USA). In the presence of 10 mM Y-27632, cells were seeded at appropriate densities (1.4 or 5.5 × 10^3^ cells/cm^2^) on iMatrix-511 or Matrigel surfaces. The cells were also seeded at 1.4 × 10^3^ cells/cm^2^ for iMatrix-511, and at 5.5–11 × 10^3^ cells/cm^2^ for Matrigel for routine maintenance. On days 1, 2, 5, and 6, the spent medium was replaced with fresh medium, and the cells were passaged on day 7. Alkaline phosphatase (ALP) staining was conducted using a kit according to the manufacturer’s instructions. Undifferentiated cells are stained in red. Colonies consisted of all undifferentiated cells (A), and colonies which contains differentiated cells (B) are counted by microscopy observations, and then the ratio of undifferentiated cell was calculated as (A)/(A + B). All test samples were prepared in triplicate and analyzed.

### 2.7. Statistical Analysis

All cell cultures were tested in triplicate, and significance tests were performed using the Student’s *t*-test in Microsoft Excel. Differences with *p* values of <0.05 were considered statistically significant.

## 3. Results

### 3.1. Effects of Test Materials on bFGF Stabilization

E8 medium was used to evaluate materials based on bFGF levels before and after warming. This medium is chemically defined and has basic components; the initial concentration of bFGF was 100 ng/mL. ReproFF culture medium was also initially used to measure the level of bFGF before and after warming. However, the E8 medium had a higher initial concentration of bFGF than ReproFF; hence, the magnitude of decrease in the level of bFGF was relatively greater in E8 medium.

**Figure 1:**
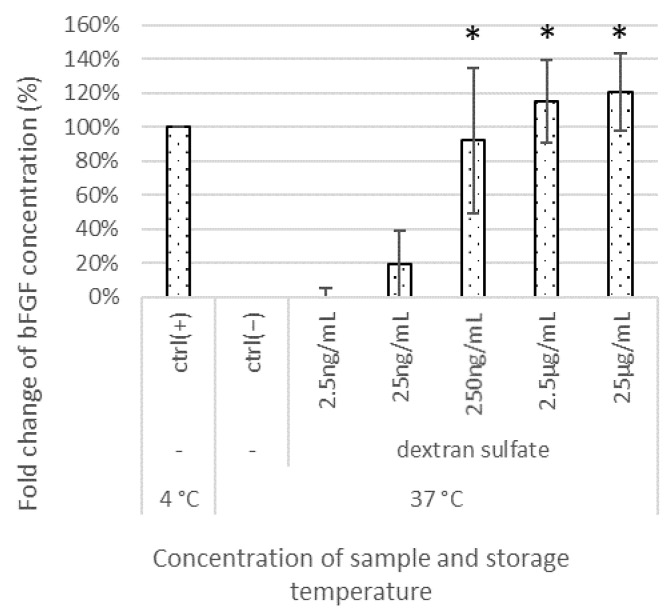
Effects of dextran sulfate on bFGF concentrations in iPSC culture media. bFGF concentrations were measured using an ELISA. The differences between the detected amounts of bFGF in test culture and negative control were considered significant at * *p* < 0.05. Microsoft Excel for Microsoft Apps for Enterprise was used for preparation of the figure.

The bFGF levels were measured after supplementing the E8 medium with different concentrations (250 pg/mL–25 μg/mL) of the test compounds (dextran sulfate, heparin, xanthan gum sulfate, fucoidan, alginate sulfate, pectin sulfate, cellulose sulfate, maltoheptaose sulfate, maltotriose sulfate, sucrose octasulfate, PVA sulfate, polyvinylamine sulfonate, polyallylamine sulfonate, and polyvinyl sulfonate) and storing the media at 37 °C for 1 week. The remaining bFGF percentage relative to the bFGF concentration in the medium stored at 4 °C (ctrl(+)) was determined. At concentrations as low as 250 ng/mL, dextran sulfate showed a positive effect, and at higher concentrations, the stabilization effect was significantly enhanced compared with that in the negative control (ctrl(−)), which was stored at 37 °C for 1 week without sulfated materials (Figure 1).

**Figure 2:**
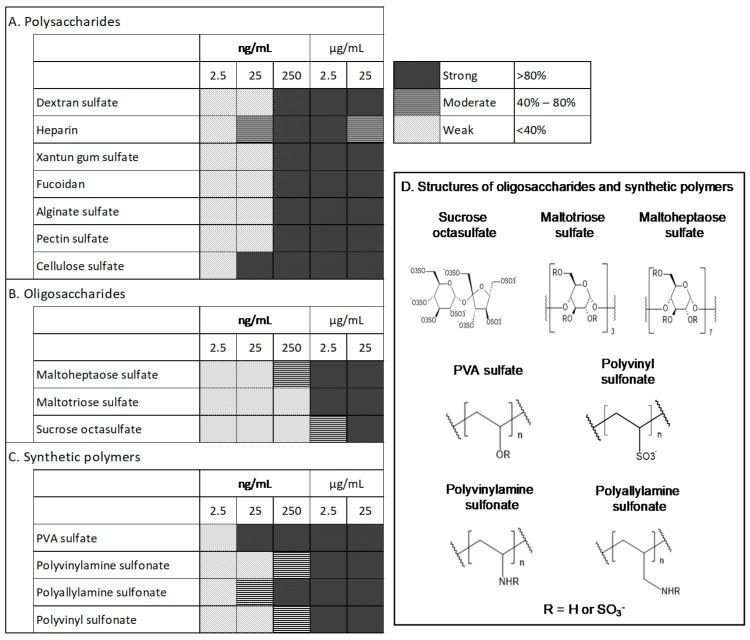
Effects of sulfated/sulfonated compounds on bFGF stabilization. Based on ELISA, as shown in Figure 1, the effective concentrations of sulfated/sulfonated polysaccharides (**A**), oligosaccharides (**B**), and synthetic polymers (**C**) for bFGF stabilization in E8 medium are shown. The structures of the sulfated/sulfonated oligosaccharides and synthetic polymers tested are also shown (**D**); R indicates H or SO_3_^−^. Samples were prepared in triplicate and analyzed. Microsoft Excel for Microsoft Apps for Enterprise was used for preparation of the figure.

When dextran sulfate was added at 25 ng/mL, 250 ng/mL, 2.5 μg/mL, and 25 μg/mL to the medium, the average percentages of the remaining bFGF relative to the initial bFGF concentration in the medium were 19%, 92%, 115%, and 121%, respectively. The results for other sulfated/sulfonated materials are summarized in Figure 2A–C. For example, when heparin was added at 25 ng/mL, 250 ng/mL, 2.5 μg/mL, and 25 μg/mL to the medium, the average percentages of the remaining bFGF relative to the initial bFGF concentration in the medium were 46%, 85%, 83%, and 58%, respectively; the average percentages with sucrose octasulfate addition were 3%, 7%, 43%, and 83%, respectively; and the average percentages with PVA sulfate addition were 113%, 131%, 136%, and 133%, respectively. We grouped the tested materials into the following three levels based on the amount of remaining bFGF: strong (>80%), moderate (40–80%), and weak (<40%). Sulfated/sulfonated polysaccharides and synthetic polymers were generally effective at 250 ng/mL. Heparin and cellulose sulfate were more effective at lower concentrations (25 ng/mL) than others, and heparin tended to be less effective at higher concentrations (25 μg/mL). To ensure efficacy, sulfated oligosaccharides were required at higher concentrations (250 ng/mL to 2.5 μg/mL) than those of sulfated polysaccharides, and the extent of the effects correlated with the chain length. Maltoheptaose sulfate, comprising seven maltose molecules, which has a longer chain than maltotriose sulfate and sucrose octasulfate, showed the highest efficacy among the oligosaccharides. Notably, among the synthetic polymers, PVA sulfate showed considerable efficacy at the lowest concentration (25 ng/mL). During the evaluation, the pH of the tested media did not change significantly.

### 3.2. Effect of Test Materials on Cell Proliferation

**Figure 3:**
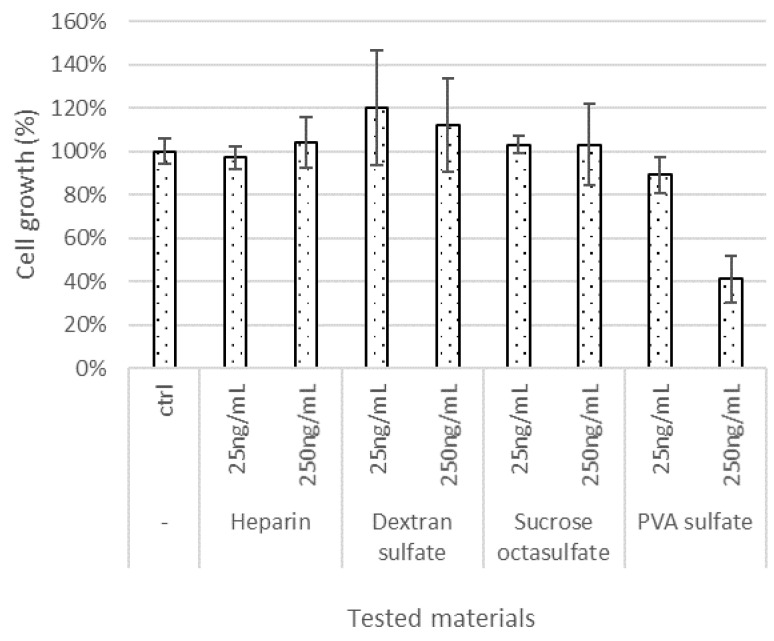
Effects of sulfated compounds on iPSC proliferation. The percentage cell growth observed for each test material at different concentrations is shown. Microsoft Excel for Microsoft Apps for Enterprise was used for the preparation of the figure.

**Figure 4:**
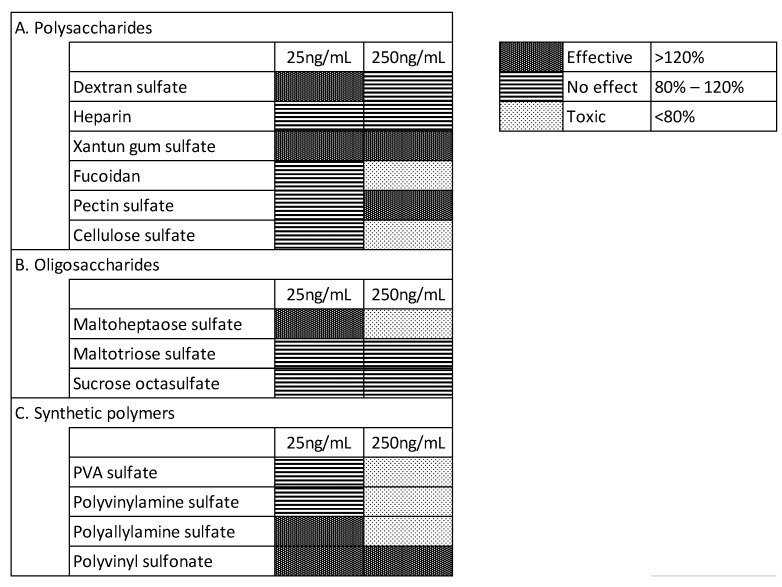
Effects of the different types of sulfated/sulfonated compounds on iPSC proliferation. Based on data obtained by the same method as in Figure 3, the effects of polysaccharides (**A**), oligosaccharides (**B**), and synthetic polymers (**C**) on iPSC culture in E8 medium with Matrigel at 25 and 250 ng/mL were assessed. The percentage cell proliferation caused by each type of material is indicated. Microsoft Excel for Microsoft Apps for Enterprise was used for the preparation of the figure.

To confirm the effects of the sulfated/sulfonated materials on iPSC cultures, we tested their effects on iPSC proliferation in the E8 medium after 7 days of continuous culture. The iPSCs were seeded in 24-well microplates (20,000 cells/well) and cultured with different test materials, which were added to E8 medium. The iPSC counts were determined on day 6. The sulfated/sulfonated materials were added to the culture medium for immediate cultivation. Matrigel was used as the scaffold. We used the materials at concentrations of 25 and 250 ng/mL, as ELISA results had shown that the lowest effective concentration was 25 ng/mL, and we observed some cytotoxicity at higher concentrations during preliminary evaluations. The results for heparin, dextran sulfate, sucrose octasulfate, and PVA sulfate are shown in Figure 3, along with the control, to which sulfated materials were not added (ctrl), and those for other materials are shown in Figure 4. We classified the tested materials into the following three levels based on the change in cell proliferation with respect to the control without any sulfonated/sulfated material: effective (>120%), no effect (120–80%), and toxic (<80%). At 25 ng/mL, some materials, such as dextran sulfate, xanthan gum sulfate, maltoheptaose sulfate, polyallylamine sulfonate, and polyvinyl sulfonate, were effective for cell proliferation. Dextran sulfate, xanthan gum sulfate, maltoheptaose sulfate, polyallylamine sulfonate, and polyvinyl sulfonate were effective even at concentrations that were found to be ineffective for bFGF stabilization, as measured by ELISA. At 250 ng/mL, xanthan gum sulfate, pectin sulfate, and polyvinyl sulfonate were effective for both cell proliferation and bFGF stabilization, whereas dextran sulfate and heparin were ineffective for cell proliferation even at concentrations found effective for bFGF stabilization.

### 3.3. Effects of the Test Materials on Medium Performance after Storage at 37 °C for 1 Week

**Figure 5:**
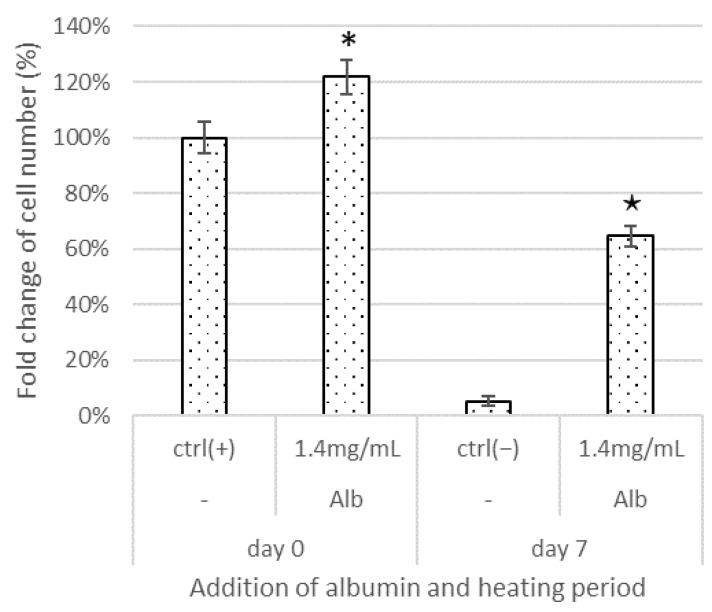
Effects of albumin and thermal treatment on iPSC proliferation. The fold-changes of cell numbers are shown. Differences between the cell numbers of test culture and positive control and negative control were considered significant at *, ^★^ *p* < 0.05, respectively. Alb: albumin. Microsoft Excel for Microsoft Apps for Enterprise was used for preparation of the figure.

Next, we evaluated whether the deterioration of the performance of the medium due to warming or storage could be suppressed by adding bFGF-stabilizing sulfated materials. We performed cell proliferation assays on media treated with these materials and then warmed them at 37 °C for 1 week. We found that the cells did not grow in the E8 medium stored at 37 °C for 1 week (Figure 5, ctrl(−)), even following the addition of the test materials (data not shown). Albumin, known to have antioxidant buffering effects, is usually added to cell culture media. One of the characteristics of the E8 medium is that it is albumin free; however, other stem cell culture media usually contain albumin. Hence, cell proliferation was further evaluated using a medium with 1.4 mg/mL albumin and warmed at 37 °C. The iPSCs were seeded in 24-well microplates (20,000 cells/well) on Matrigel. The iPSC counts were determined on day 6. As shown in Figure 5, when albumin was added to the E8 medium (Alb, day 0), cell growth was significantly promoted compared with that in the positive control (ctrl (+)), which was cultured in the medium prepared at the time of use without albumin. In addition, even after storage at 37 °C for 1 week (Alb, day 7), significant cell proliferation was observed in the medium with albumin compared to that in the negative control (ctrl(−)), which was cultured in the medium without albumin; however, the proliferation was lower than that observed in the ctrl (+).

**Figure 6:**
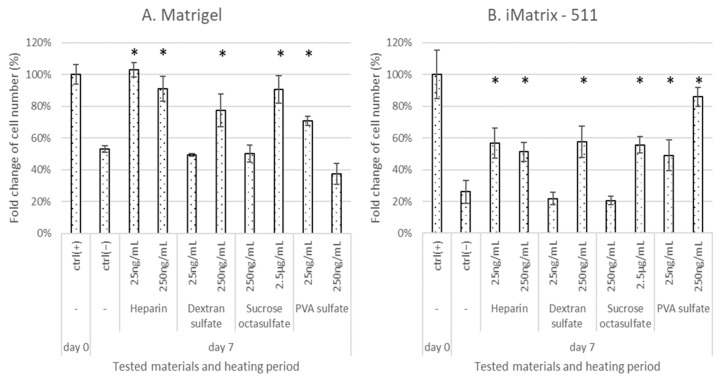

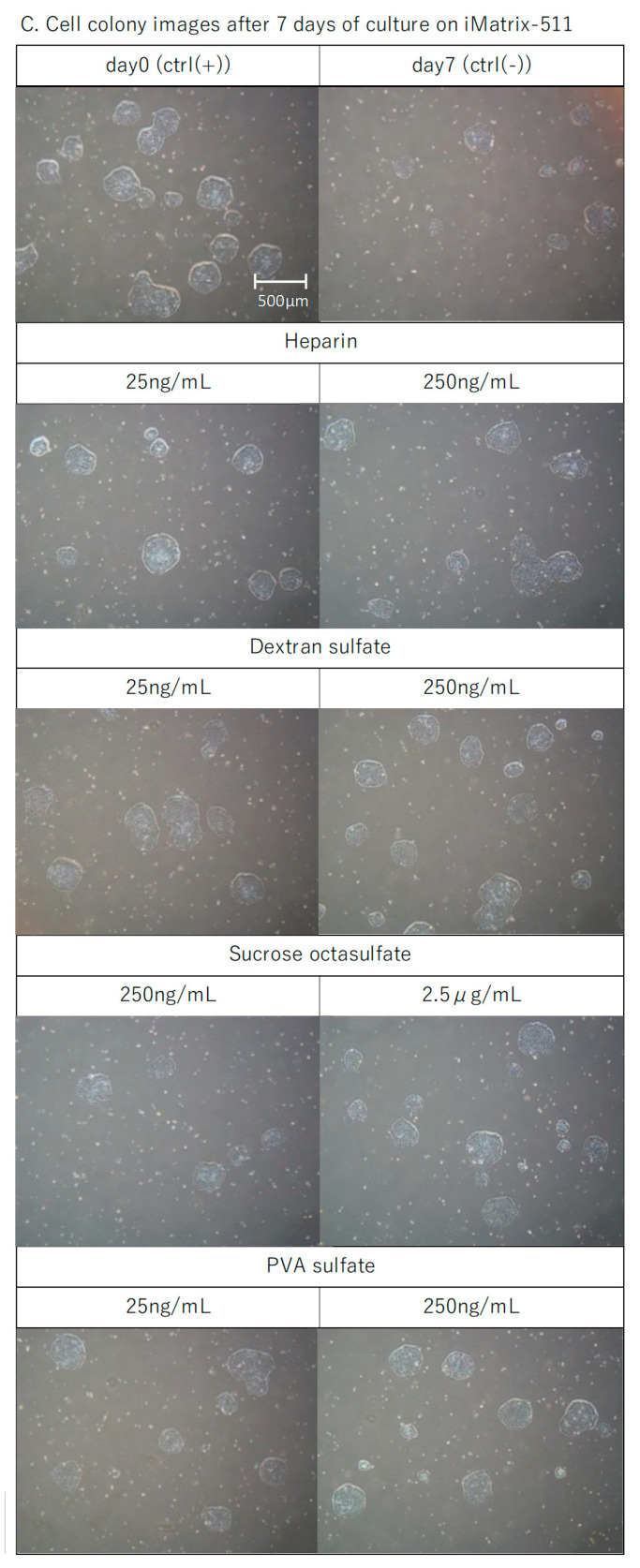
Effects of sulfated materials and heating of cell culture media on iPSC proliferation on Matrigel (**A**) or iMatrix-511 (**B**). The fold-changes in cell proliferation are shown. Differences between the cell numbers of test and negative controls were considered significant at * *p* < 0.05. Cell colony images after 7 days of culture on iMatrix-511 are shown (**C**). Microscopy images and colony counts were obtained using the Keyence BZ-X710 fluorescence microscope and its BZ-X Analyzer software (Osaka, Japan). Microsoft Excel for Microsoft Apps for Enterprise was used for preparation of the figure.

Based on this observation, we systematically evaluated the effects of sulfated materials on heat-induced deterioration of albumin-added E8 medium. The culture in medium that was not stored was used as the positive control (ctrl(+)), and that in the stored medium was used as the negative control (ctrl(−)). Heparin, dextran sulfate, sucrose octasulfate, and PVA sulfate were used for the evaluation because of their structural features as polysaccharides, oligosaccharides, and synthetic polymers and their ease of availability. E8 medium containing albumin supplemented with 25 or 250 ng/mL sulfated materials was warmed at 37 °C for 1 week, and then used for iPSC culture. Sucrose octasulfate was added at 250 ng/mL or 2.5 μg/mL because it was effective exclusively at higher concentrations for maintaining bFGF levels. Matrigel and iMatrix-511 were chosen as they are popular and chemically defined scaffolds, respectively. The iPSCs were seeded in 24-well microplates (20,000 cells/well). The iPSC counts were determined on day 6. As shown in Figure 6A, when Matrigel was used as a scaffold, cell growth in the stored medium was about half of that in the ctrl(+). In the warmed culture media, heparin at both levels (25 and 250 ng/mL), dextran sulfate at 250 ng/mL, PVA sulfate at 25 ng/mL, and sucrose octasulfate at 2.5 μg/mL significantly suppressed the inhibition of the cell proliferation observed in the ctrl(−), with cell proliferation recovering to a level close to that of the ctrl(+). However, PVA sulfate suppressed cell growth at a concentration of 250 ng/mL. The results obtained with iMatrix-511 as a scaffold were similarly evaluated (Figure 6B). Similar to Matrigel, the medium warmed at 37 °C for 1 week (ctrl(−)) showed reduced cellular growth, but the level was greater than that observed when Matrigel was used. Heparin, dextran sulfate, and sucrose octasulfate significantly suppressed the decrease in cell proliferation with iMatrix-511 at the same concentrations as were observed with Matrigel. Unlike with Matrigel, PVA sulfate suppressed the decline in cell proliferation in a dose-dependent manner with iMatrix-511. None of the test materials caused abnormally shaped colonies (Figure 6C). Materials that significantly suppressed cell growth reduction with iMatrix-511 also resulted in an increased number of colonies, which mean increased cell proliferation. The ratio of undifferentiated cells measured by alkaline phosphatase (ALP) staining was approximately the same as that observed for the ctrl(+) when sulfated materials were added to the medium and cultured with iMatrix-511 (98–100%).

## 4. Discussion

In this study, we showed that sulfated/sulfonated polysaccharides and synthetic polymers generally exhibited strong bFGF-stabilization effects at 250 ng/mL (Figure 2). The concentrations of sulfated oligosaccharides with bFGF-stabilizing effects varied in a chain length-dependent manner. The affinity of sulfated polysaccharides to bFGF is considerably higher than that of oligosaccharides due to their higher molecular weight and abundance of sulfate groups. Moreover, PVA sulfate differs from polysaccharides in its main backbone structure. Therefore, PVA sulfate might have a higher affinity for bFGF, which could explain its efficacy at lower molar levels.

When cultured in media with added sulfated/sulfonated materials used immediately after preparation, cell growth was enhanced (Figure 3 and Figure 4). Some test materials (dextran sulfate, xanthan gum sulfate, maltoheptaose sulfate, polyallylamine sulfonate, and PVA sulfate) exhibited this effect at 25 ng/mL. The effective concentration in the ELISA experiment was higher for most materials other than PVA sulfate. The difference in the concentration effective for cell growth and bFGF stabilization may be attributed to the low efficacy of materials that were not detected in the ELISA experiment, which measures only the immunoreactive molecular species of bFGF. Alternatively, sulfated materials may not only protect bFGF but also affect other factors such as cell physiology and culture environment. Half of the tested materials were toxic at high concentrations and inhibited the proliferation of iPSCs. This toxicity may be attributed to the molecular structure of the materials, the number of sulfate groups, molecular size, or impurities derived during the synthesis.

The performance of the E8 medium deteriorated and cell growth was inhibited at a medium temperature of 37 °C. The depletion of bFGF in media after 1 week of warming was confirmed using ELISA (Figure 1), which may be among the reasons for the deteriorated performance. Albumin is essential for serum-free culture [32], and in contrast to other iPSC culture media, the E8 medium is albumin-free [33]. The addition of albumin to the E8 medium improved its performance and significantly promoted cell proliferation, especially after storage at 37 °C for 1 week. Such effects could be attributed to its antioxidant properties in cell culture environments [34] or to other factors such as the change in osmotic pressure. However, the bFGF concentration measured via ELISA was depleted after storage at 37 °C, irrespective of the addition of albumin (data not shown). Hence, the presence of albumin is unlikely to affect bFGF stabilization.

When the E8 medium supplemented with albumin and sulfated materials and warmed at 37 °C for 1 week was used for culture, cell growth was significantly enhanced (Figure 5). The sulfated material stabilized bFGF when added to the medium, but both albumin and the sulfated material were required to restore the performance of the medium deteriorated by heating. Although these showed additive effects, albumin helped restore the performance of the medium deteriorated by heating when added alone, whereas the sulfated materials did not have such effect, which suggests that the effects of albumin and sulfated materials have different mechanisms; albumin seems to protect the medium from other deteriorating factor, which is caused by heating. Further research on the effects of albumin and research on alternative materials of albumin are necessary to optimize culture conditions.

The four tested materials (heparin, dextran sulfate, sucrose octasulfate, and PVA sulfate) were effective at some concentrations with both Matrigel and iMatrix-511 used as scaffolds (Figure 6). The effective concentrations for ELISA experiments were similar to those for cell proliferation. The bFGF concentration in the medium supplemented with albumin and sulfated materials, warmed at 37 °C, and stored for 1 week was as high as that observed before warming the medium. The degree of efficacy shown using ELISA (‘strong’ or ‘moderate’) did not affect cell proliferation; therefore, bFGF stabilization above a threshold can be effective for cell proliferation. In our previous study, the proliferation of human mesenchymal cells was lower when the cells were cultured with deteriorated media, and cell proliferation recovered when bFGF was supplemented in such media [21]. Similarly, stabilizing bFGF in deteriorated media can enhance hiPSC proliferation. However, further studies are warranted in order to ascertain whether the observed effects of sulfated materials are attributable only to the stabilizing action on bFGF, as sulfated materials are known to interact with extracellular matrices [35], and other biomolecules.

The undifferentiated ratio of cells after cell culture with tested materials measured by ALP staining was as same level as that of ctrl(+), and colonies maintained a circular shape similar to that of the ctrl(+). These indicators are regarded as good references to examine the undifferentiated status of iPS cells, respectively [36,37]. Hence, iPS cells are considered to maintain undifferentiated status. However, this result does not provide sufficient information on cell status, further studies are warranted to investigate it in detail, where undifferentiated markers or morphology analysis can be considered.

The tested materials suppressed the heat-induced decline of bFGF concentration, which lends support to the concept of suppressing heat-induced medium deterioration.

Culture media with Matrigel scaffolds, when warmed without sulfated materials (ctrl(−)), lost approximately 50% of their performance, compared to ctrl(+), whereas those with iMatrix-511 lost 70% of their performance. In media with sulfated materials, cell growth was restored to more than 80% of ctrl(+) with Matrigel, but only up to 60% of ctrl(+) with iMatrix-511, except for the treatment with 250 ng/mL PVA sulfate. Heat-induced decrease in medium performance was suppressed by approximately 30% by the addition of sulfated materials on both scaffolds, but the impact of warming varied for the two scaffolds. The highly purified laminin 511-E8 fragments with an integrin-binding sequence of iMatrix-511 related to the specialization of its function for adhesion to iPSCs [38]. In contrast, Matrigel is composed of complex materials of animal origin [39]. This difference in the compositions of the scaffolds may have affected the results. With PVA sulfate, cell proliferation was inhibited at a high concentration on Matrigel; however, it was enhanced on iMatrix-511 in a dose-dependent manner. The sulfated materials may have some roles other than stabilizing bFGF. Further studies are warranted to ascertain how sulfated materials interact with not only bFGF but also cells or scaffolds and how these interactions affect the cell culture environment or cell proliferation. The effect of pluronic copolymers on stem cell culture has been reported [40], and it is similar to the effect of some other synthetic polymers. Pluronic comprises block copolymers of polyoxyethylene–polyoxypropylene–polyoxyethylene, and these copolymers, with appropriate molecular lengths and component ratios, can enhance the pluripotency of the stem cells. Therefore, polymers with appropriate chemical properties can confer beneficial effects on stem cells. PVA sulfate may have hydrophobic and ionic interactions with the cell surface or scaffolds, which may contribute to the observed cytotoxicity with Matrigel and cell proliferation with iMatrix-511. The sulfur content of the PVA sulfate tested was 15%, and approximately, 40% of the hydroxyl residues were sulfated. These differences may be reduced by balancing the ionic residues and hydrophobicity by adjusting the number of sulfate groups in the molecule.

Further investigation, including the effect of longer period and cell characteristics, is needed to clarify the underlying mechanism of toxicity to cell proliferation to characterize toxic materials.

## 5. Conclusions

We reported the potential of a novel and effective iPSC culture method involving the addition of easily available and simple chemically defined sulfate materials to culture medium in the presence of albumin. We demonstrated that this method suppresses the heat-induced decline in bFGF levels, which in turn suppresses the deterioration of the medium during storage. The addition of chemically defined and easily available materials, such as dextran sulfate, sucrose octasulfate, and PVA sulfate, to the culture medium also meets the requirement of maintaining xeno-free conditions in cell culture for applications in regenerative medicine. Therefore, this method could potentially be applied in automated culture or 3D culture for practical realization.

## Data Availability

The data presented in this study are available on request from the corresponding author. The data are not publicly available due to commercial restrictions.
